# Minimum inhibitory concentration of nano-silver bactericides for beneficial microbes and its effect on *Ralstonia solanacearum* and seed germination of Japanese Cucumber (*Cucumis sativus*)

**DOI:** 10.7717/peerj.6418

**Published:** 2019-03-20

**Authors:** Poopak Sotoodehnia, Norida Mazlan, Halimi Mohd Saud, Wahid A. Samsuri, Sheikh Hasna Habib, Amin Soltangheisi

**Affiliations:** 1 Department of Agriculture Technology, Universiti Putra Malaysia, Selangor, Malaysia; 2 Department of Land Management, Faculty of Agriculture, Universiti Putra Malaysia, Selangor, Malaysia; 3 Oil Seed Research Center, Bangladesh Agricultural Research Institute, Joydebpur, Bangladesh; 4 Center of Nuclear Energy in Agriculture (CENA), University of Sao Paulo, Sao Paulo, Brazil

**Keywords:** Nanoparticles, PGPR, Seed germination

## Abstract

**Background:**

Plant growth-promoting rhizobacteria (PGPR) are highly promising biofertilizers that contribute to eco-friendly sustainable agriculture. There have been many reports on the anti-microbial properties of nanoparticles (NPs). Toxic effects of NPs under laboratory conditions have also reported; however, there is a lack of information about their uptake and mobility in organisms under environmental conditions. There is an urgent need to determine the highest concentration of NPs which is not detrimental for growth and proliferation of PGPR.

**Methods:**

Transmission electron microscopy (TEM) and scanning electron microscopy (SEM) were used to measure the size and shape of NPs. Minimum inhibitory concentrations (MIC) of nano-silver on selected beneficial microbes and *Ralstonia solanacearum* were measured using the microdilution broth method. The percentage of seed germination was measured under *in vitro* conditions.

**Results:**

NPs were spherical with a size of 16 ± 6 nm. Nano-silver at 12–40 mg l^−1^ inhibited the growth of bacteria. Seed application at 40 mg l^−1^ protected seeds from *R. solanacearum* and improved the rate of seed germination.

## Introduction

*Ralstonia solanacearum* is a soil borne bacterial pathogen identified as one of the major limiting factors for the production of many crops around the world. It causes the widespread devastating disease, known as bacterial wilt, in more than 450 potential host plant species, such as potato, tomato, eggplant, cucumber, ginger, tobacco and banana. This pathogen enters through the roots and reaches the xylem, from where it spreads through the plant. As it infects the plant vascular system, it can lead to death of the entire plant (Deberdt et al., 2014). Some bacteria strains have become resistant to the antibacterial agents due to their continuous application around the world, especially on agricultural land. Increasing use of pesticides affects non-target species and also pollutes water bodies, influencing the environment and human health. Using new approaches like nanotechnology to reduce the negative impact of pesticides on the environment is crucial (Ericksen, Ingram & Liverman, 2009). Among metal nanoparticles (NPs), nano-silver (Ag) is being used in agriculture as a bactericide and fungicide due to its high antibacterial and antifungal properties (Sotiriou & Pratsinis, 2011).

In order to enhance the effectiveness of silver NPs, the ratio of surface area to volume is very important. Higher ratios enhance the antibacterial effects due to greater interaction with other particles (Lee & Meisel, 1982; Lkhagvajav et al., 2011). The toxic effect of NPs on bacteria largely depends on the type and size of NPs and on the type of bacteria. Beneficial microbes are responsible for multiple biochemical processes in soil, such as nutrient mineralization, nitrogen cycling and organic carbon degradation, and they also play a key role in biological cycling of nutrients (Holden, Schimel & Godwin, 2014). Nano-silver should be applied to seeds or plants at levels that control plant disease without any harmful impact on beneficial microbes. Metal oxides, known as inorganic NPs, could inhibit bacterial activity even at very low concentrations (<1 mg kg^−1^; Simonin & Richaume, 2015). Among metal NPs, silver influenced bacterial activity, even at a very low concentration (3.2 µg kg^−1^; Colman et al., 2013). Hao et al. (2017) studied the antifungal effects of 6 NPs, multi-walled carbon nanotubes (MWCNTs), fullerene (C60), reduced graphene oxide (rGO), copper oxide (CuO), ferric oxide (Fe_2_O_3_) and titanium oxide (TiO_2_) on *Botrytis cinerea.* He found that all 6 NPs could inhibit the growth of *B.cinerea* and this inhibitory effect highly depends on the concentration and type of NPs (Hao et al., 2017).

Many studies have been conducted to measure minimum inhibitory concentrations (MICs) of NPs for different bacteria. MICs of AgNPs for *E.coli*, *Listeria monocytogenes*, *Salmonella typhimurium*, and *Vibrio parahaemolyticus* were within the range 3.12–6.25 µg ml^−1^ (Zarei, Jamnejad & Khajehali, 2014), while MICs of nano-silver for *Pseudomonas aeruginosa*, *Bacillus subtilis*, *Candida albicans*, and *Staphylococcus aureus* were within the range 2–4 µg ml^−1^ (Lkhagvajav et al., 2011). Jung et al. (2010) showed that nano-silver application at 7 mg Ag ml^−1^ could inhibit the activity of *Sclerotium cepivorum*, a fungus which causes white rot disease in green onions.

In commercial agriculture, rapid and uniform seed germination and seedling emergence are important factors in successful stand establishment (Hojjat & Hojjat, 2015; Rui et al., 2016; Yang, Cao & Rui, 2017). In many studies, appropriate dosing of NPs can promote seed germination, improve soil quality, decrease the residue of pesticides, protect seeds from pathogens and promote plant growth, without any influence on beneficial microbes (Khodakovskaya et al., 2009; Rui et al., 2018; Yang, Cao & Rui, 2017).

The objectives of this study were to determine the MIC of nano-silver for beneficial microbes in soil. In addition, we evaluated the effect of nano-silver applied to the seeds of *Cucumis sativus*, as a bactericide against *R. solanacearum* and in promoting seed germination. It is noteworthy that different areas of the world have different types of beneficial microbes. This study is the first investigating the effects of AgNPs on beneficial microbes in Malaysia.

## Materials & Methods

Commercial nano-silver (colloidal Ag) bactericide was purchased from Nano Nasb Pars Co, Tehran, Iran, at an initial concentration of 4,000 mg kg^−1^. It was purified by the company according to their production manual. For this study, bacterial isolates UPMR bio1 and UPMR bio2 were isolated from Alkazot bio fertilizer (Alkan Co., Tehran, Iran). Other beneficial bacteria and *R. solanacearum* were purchased from the Microbial Technology Laboratory, University Putra Malaysia (UPM). Triphenyl tetrazolium chloride (TTC; Sigma-Aldrich, St. Louis, MI, USA) was used as an indicator to measure microbial respiration. Nutrient broth (NB) was purchased from Merck, Darmstadt, Germany. Japanese cucumber (*C. sativus*) seeds hybrid 309 (Green World Genetics Company, Kuala Lumpur, Malaysia) were used in this study.

### Characterization of nano-silver bactericide

The morphology of the samples was determined by scanning electron microscopy (SEM), at the Institute of Bioscience, UPM. For this purpose, 1 drop of nano-silver was placed on aluminum foil and dried at room temperature. Shape and morphology of AgNPs were investigated with a JSM-7600F SEM (JEOL, Tokyo, Japan) at an accelerating voltage of 15 kV and magnifications up to 50,000X. The transmission electron microscopy (TEM) at the Faculty of Medicine, University of Malaya (UM), Kuala Lumpur, Malaysia was used to measure the size distribution of AgNPs. The TEM was operated at an accelerating voltage of 120 kV. For this study, 1 ml AgNPs was diluted in 80 ml of distilled water. This mixture was homogenized by sonication in a Sonorex, RK 100H (Bandelin, Berlin, Germany) for 30 min. One drop of this preparation of AgNPs was then placed on each carbon-coated copper grid and left for 48 h to dry.

### MIC of colloidal nano-silver for selected bacteria

The MIC was defined as the lowest concentration of an antibacterial agent which inhibited the activity and growth of a microorganism after overnight incubation. A broth microdilution method was used to measure the MIC of colloidal nano-silver. Bacteria for this study were selected based on their classification (Gram-negative and Gram-positive) and their availability in the Microbial Technology Laboratory. The bacteria tested in this study were: UPMR1004, *Paenibacillus sp;* UPMR 040, *Pseudomonas sp;* UPMR Sm1, *Bacillus pumilus;* UPMR Skb1, *Acinetobacter brisouii;* UPMR Skb2, *Bacillus pumilis;* UPMR Skb7, *Bacillus cereus;* UPMR Skb10, *Chromobacterium violaceum;* UPMR 9, *Burkholderia sp;* UPMR rubber, *Enterobacter sp;* UPMR bio1, *Citrobacter amalonaticus;* UPMR bio2, *Citrobacter farmeri;* UPMR 14, an unidentified isolate; UPMR 1021, *Bacillus cereus*; and *R. solanacearum*. Microbial inocula were prepared by subculturing microorganisms in NB at 37 °C for 24 h. A nano-silver stock solution (40 mg l^−1^) was prepared with ultrapure distilled water. A 2 mg l ^−1^ incremental dilution series of nano-silver was made in the range of 2–40 mg l^−1^. A 100 µl aliquot of each nano-silver concentration and 100 µl of NB containing the tested microorganisms were added to wells of a microplate. The microplates were then incubated at 37 °C for 24 h. After incubation, 40 µL of 0.02 mg ml^−1^ TTC was added to each well. Color change of TTC from colorless to red was considered a positive indication of microbial growth (Lkhagvajav et al., 2011, with minor modifications).

### Seed preparation

The surfaces of the seeds were sterilized by using 0.5% (v/v) sodium hypochlorite for 20 min, and then they were rinsed several times with deionized water (Habib, Kausar & Saud, 2016). As a control (T1), seeds were soaked in distilled water for 48 h. Seeds in sample T2 were soaked in 40 mg l^−1^ AgNPs (based on the MIC results) for 48 h at 31 °C. For sample T3, seeds were soaked in 40 mg l^−1^ AgNPs for 48 h at 31 °C, and then kept in bio fertilizer containing bacteria UPMR bio1 and UPMR bio2 for 20 min. For sample T4, seeds were soaked in 40 mg l^−1^ nano-silver for 48 h at 31 °C, and then placed in a culture of *R. solanacearum* for 20 min. For sample T5, seeds were soaked in 40 mg l^−1^ nano-silver for 48 h at 31 °C, followed by soaking in a culture of *R. solanacearum* for 20 min, and then kept in bio fertilizer for 20 min. Finally, two moist sterilized sheets of Whatman number 2 filter paper (Whatman International Ltd, Maidstone, England) were placed in each petri dish (9 mm diameter). After the seeds had been treated they were placed in these petri dishes. Four seeds were placed in each petri dish and after 7 days, the percentage of seed germination was calculated (Table 1).

\begin{eqnarray*}\text{%Germination}= \left( \frac{\text{number of germinated seeds} }{\text{total number of seeds planted} } \right) \times 100 \quad \quad \quad (Karimi etal,2012). \end{eqnarray*}

### Statistical analysis

Experiment for measuring the percentage of seed germination was conducted using completely randomized design (CRD) with a total number of 5 treatments with 4 replications. The means were compared by Tukey’s test. Values of *p* ≤ 0.05 were considered to be statistically significant. Data were statistically analysed using SAS 9.4 software (SAS Institute, Cary, NC, USA).

## Results

### Characterization of nano-silver bactericides

**Figure 1:**
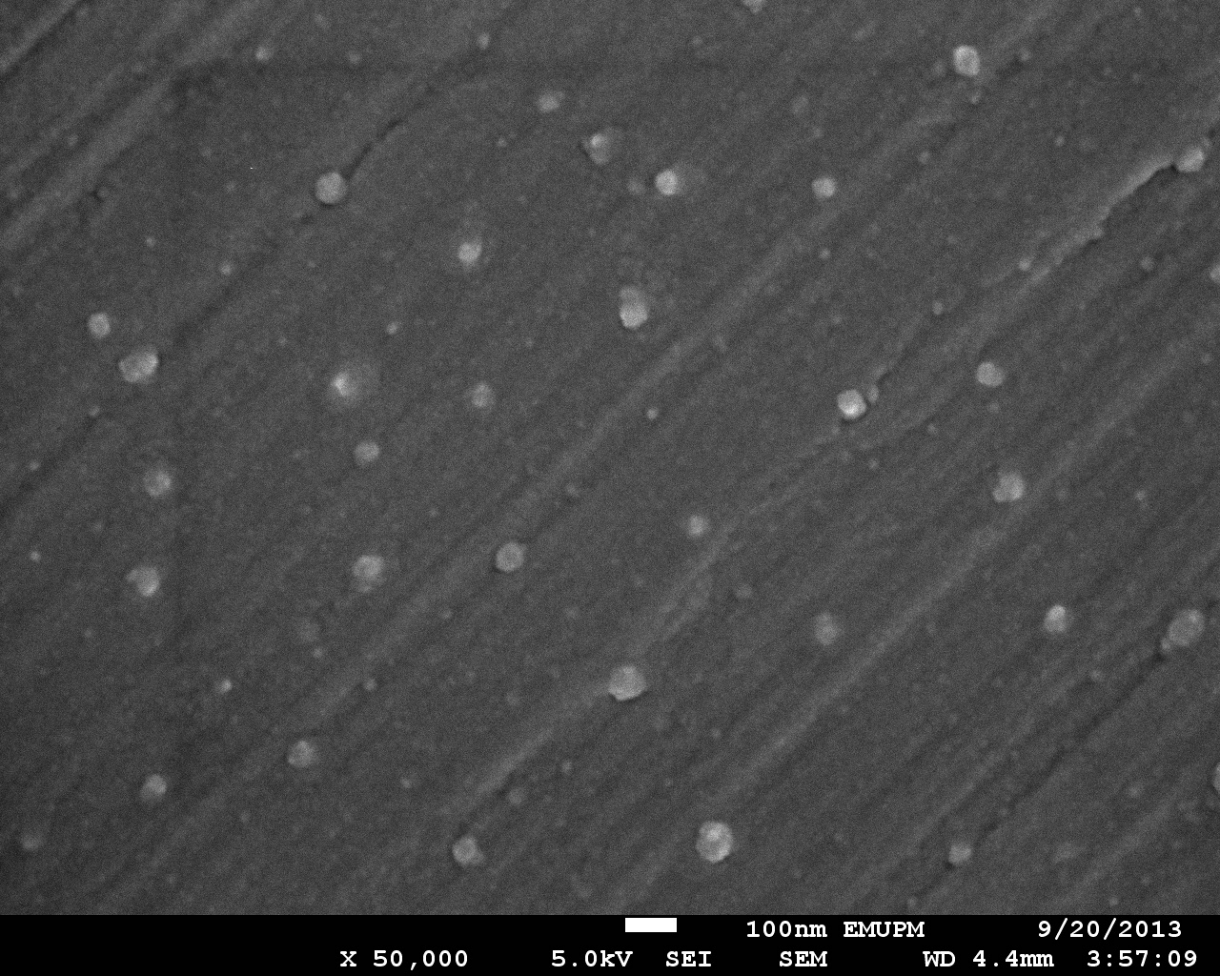
SEM image of nano-silver bactericides at 50,000 X.

SEM showed that silver NPs spherical with a smooth morphology, and were evenly distributed in solution (Fig. 1). Silver NPs were single, and did not exhibit aggregation. In addition, the purity of the samples was consistent with the claims of the manufacturer.

**Table 1:** Treatments used for evaluating seed germination.

Treatments	Description
T1	Control
T2	AgNPs
T3	AgNPs + UPMR bio1 +UPMR bio2
T4	AgNPs +*Ralstonia solanacearum*
T5	AgNPs +*Ralstonia solanacearum*+** UPMR bio1 +UPMR bio2

**Figure 2:**
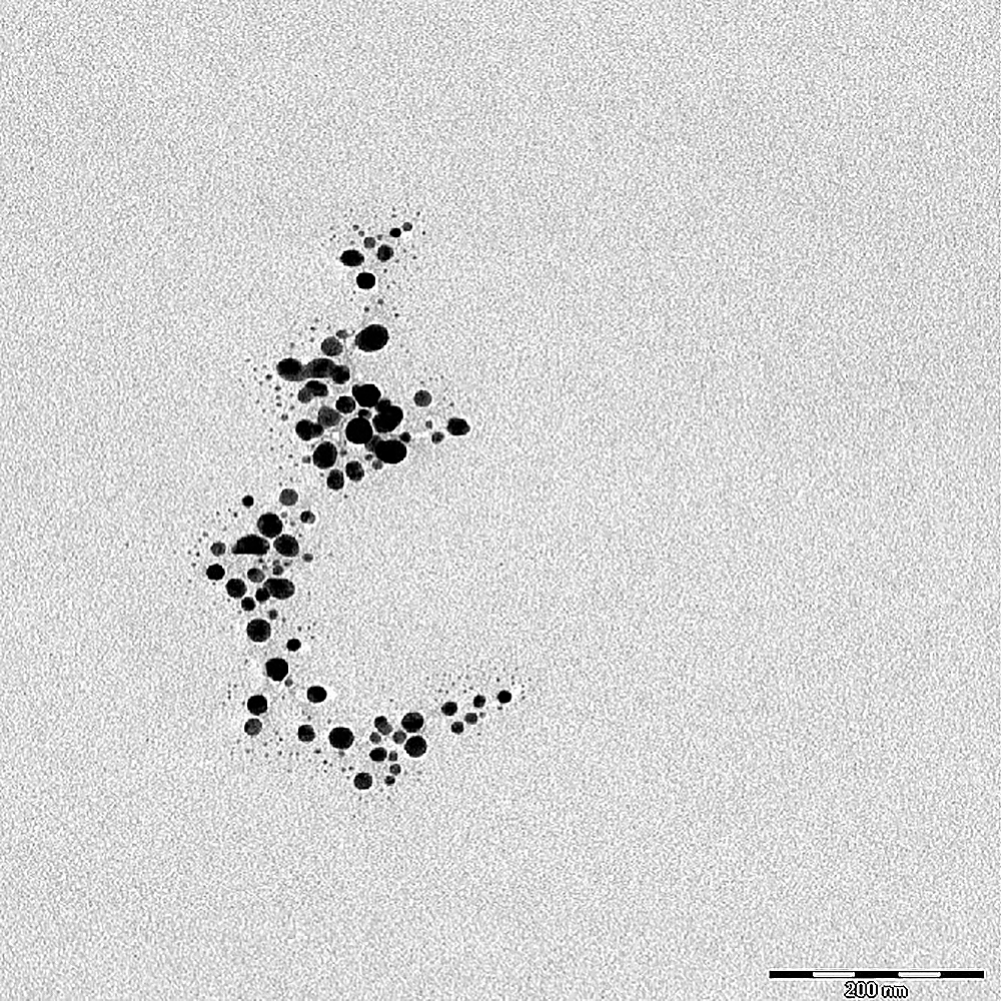
TEM image of silver NPs. Scale = 200 nm.

**Figure 3:**
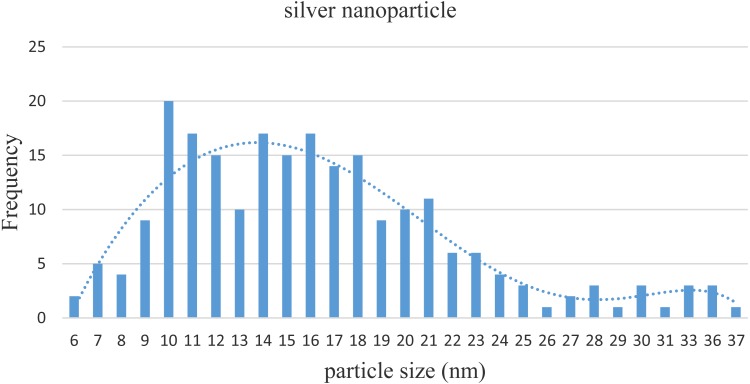
Distribution of sizes of silver NPs based on the TEM results.

Images from TEM showed that the particle sizes of nano-silver were 6–37 nm with an average size of 16 ± 6 nm (standard deviation, SD 6.20; Fig. 2). Figure 3 shows the size distribution of NPs.

### Biochemical classification of beneficial microorganisms

A total of 13 beneficial microbes were tested in the MIC experiment. The beneficial microbes used in this study were classified into two main groups, nitrogen fixing bacteria (NFB) and phosphate solubilizing bacteria (PSB). UPMR 1004, UPMR 040, UPMR Sm1, UPMR Skb1, UPMR Skb2, UPMR Skb7, UPMR Skb10, UPMR rubber, UPMR 1021 were classified as PSB, while UPMR bio1 and UPMR bio2, isolated from Alkazot bio fertilizer, were classified as NFB (Table 2). UPMR 040, UPMR 9, and UPMR 14 were classified as both NFB and PSB, meaning that these beneficial microbes had the ability to fix nitrogen and solubilize phosphate. UPMR 1004, UPMR Sm1, UPMR Skb2, UPMR Skb7, and UPMR 1021 were classified as Gram-positive bacteria, while the rest were Gram-negative.

**Table 2:** Classification of beneficial microbes used in MIC experiment. The beneficial bacteria that used for this study was classified based on their ability to fixing nitrogen and solublizing phosphate, and also based on their strain (gram negative and positive).

**Strain no**	**Name**	NFB*	PSB*	**Gram negative**	**Gram positive**
UPMR 1004	*Paenibacillus sp*		√		√
UPMR 040	*Pseudomonas* sp	√	√	√	
UPMR Sm1	*Bacillus pumilus*		√		√
UPMR Skb1	*Acinetobacter brisouii*		√	√	
UPMR Skb2	*Bacillus pumilis*		√		√
UPMR Skb7	*Bacillus cereus*		√		√
UPMR Skb10	*Chromobacterium violaceum*		√	√	
UPMR 9	*Burkholderia* sp	√	√	√	
UPMR rubber	*Enterobacter* sp		√	√	
UPMR 14	*Unidentified*	√	√		
UPMR bio1	*Citrobacter amalonaticus*	√		√	
UPMR bio2	*Citrobacter farmer*	√		√	
UPMR1021	*Bacillus cereus*		√		√

**Notes.**

*PSB Phosphate solubilizing bacteria *NFB Nitrogen fixing bacteria

### MIC of nano-silver for selected bacteria

The results from the MIC study showed that nano-silver colloids exhibit different antibacterial activities for each respective beneficial microbe tested. The MIC for the selected beneficial microbes varied from 12 to 40 mg l^−1^ (Table 3).

**Table 3:** MIC for selected beneficial microbes. Minimum inhibitory concentration of nano silver was presented on different type of bacteria that used for this study.

Strain no	Name	MIC of colloidal Nano silver (mgl ^−1^) against tested bacteria
UPMR bio1	*Citrobacter amalonaticus*	40
UPMR 040	*Pseudomonas* sp	40
UPMR bio2	*Citrobacter farmer*	40
UPMR Skb1	*Acinetobacter brisouii*	40
UMPR Skb2	*Bacillus pumilis*	40
UPMR Skb7	*Bacillus cereus*	40
UPMR Skb10	*Chromobacterium violaceum*	40
UPMR 9	*Burkholderia* sp	40
UPMR 14	*Unidentified*	40
UPMR rubber	*Enterobacter* sp	36
UPMR Sm1	*Bacillus pumilus*	28
UPMR 1004	*Paenibacillus sp*	14
UPMR1021	*Bacillus Cereus*	12

UPMR bio1 and UMPR bio2 could resist nano-silver concentrations of 40 mg l^−1^. The MIC of nano-silver for UPMR 040, UMPR Skb1, UPMR Skb2, UPMR Skb7, UPMR Skb10, UPMR 9, and UPMR 14 was 40 mg l^−1^. However, silver NPs, as a bactericide, could affect the activity of UPMR rubber at a concentration of 36 mg l^−1^, and the MIC for UPMR Sm1 was 28 mg l^−1^. UPMR 1004 and UMPR 1021 were affected by the lower concentrations of the nano-silver bactericide at levels of 14 and 12 mg l^−1^, respectively. Bacteria able to both fix N and solubilize P were more resistant to nano-silver than those which had only one of those capabilities. This resistance might be due to the thickness of their cell wall or possibly strain variation. The MIC results showed that Gram-positive bacteria were more susceptible to silver NPs than Gram-negative bacteria. *R. solanacearum* which was a Gram-negative plant pathogenic bacterium had a MIC of 40 mg l^−1^. The growth/activity of Gram-negative bacteria was inhibited at nano-silver concentrations of 36–40 mg l^−1^, while it was 12–28 mg l^−1^ for Gram-positive bacteria.

### Effect of nano-silver bactericide on seed germination

In sample T1, where the seeds were soaked in deionized water, 87.5% of them germinated (Table 4). By contrast, 100% germination was observed in samples T2, T3, and T4. For T5, the rate of germination was reduced to 75%, the lowest germination rate among all the treatments, and which was significantly lower than samples T2, T3, and T4.

**Table 4:** Percentage of seed germination for different treatments after 7 days.

Treatments	T1	T2	T3	T4	T5
Seed Germination (%)	87.5ab	100a	100a	100a	75b

**Notes.**

* Means with same and small letters in each row are not significantly different as *p* ≤ 0.05.

** (T1), Control (only deionized water); (T2), AgNPs; (T3), AgNPs + UPMR bio1 + UPMR bio 2; (T4), AgNPs + *Ralstonia solanacearum*; (T5), AgNPs + *Ralstonia solanacearum* + UPMR bio1 +UPMR bio2

**Figure 4:**
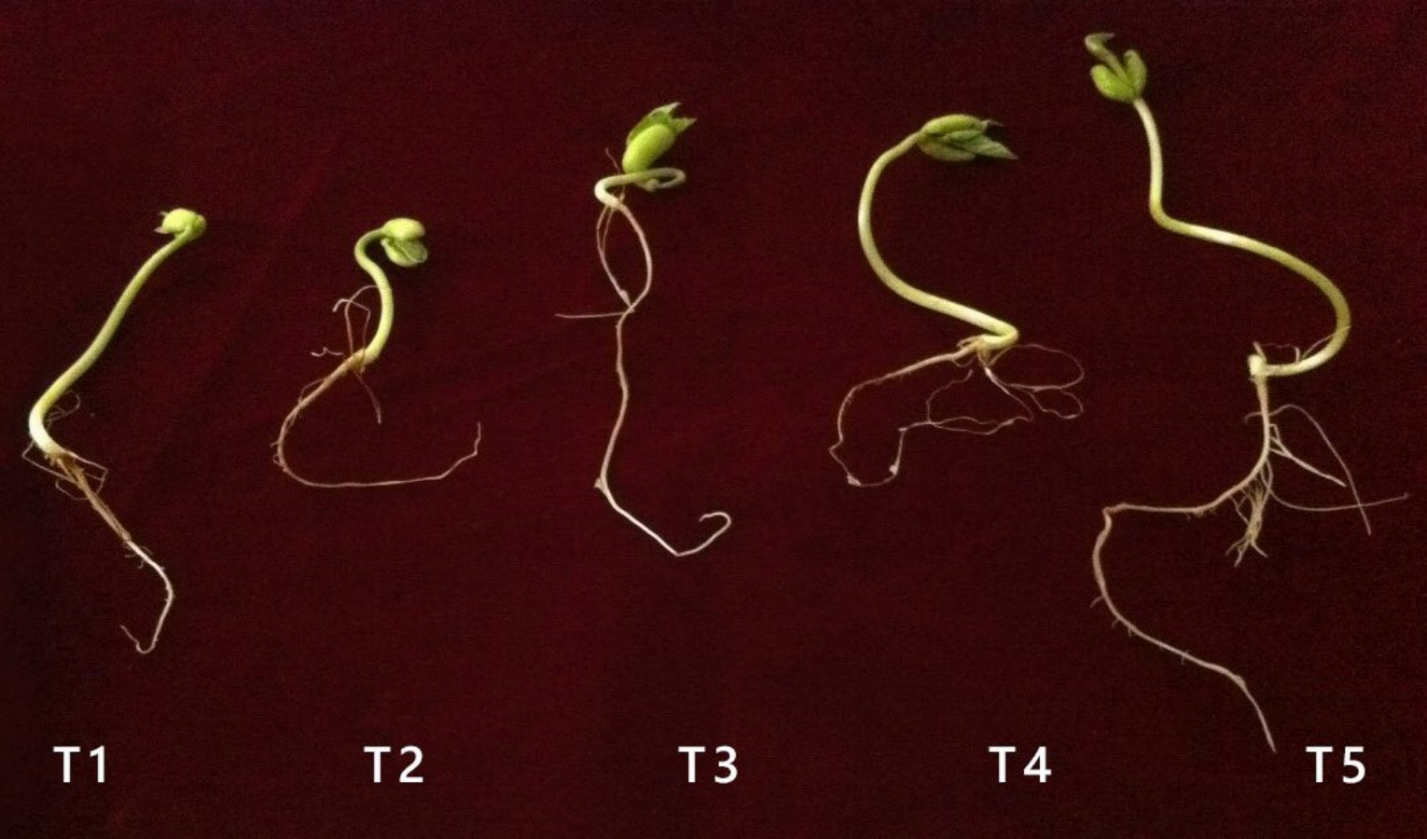
Seed germination after 7 days. Photos by Poopak Sotoodehnia.

Germination of seeds soaked in nano-silver was accelerated compared to the control, where seeds were soaked in deionized water (Fig. 4). A MIC of 40 mg l^−1^ was recorded for UPMR bio1 and UPMR bio2, which were beneficial microbes and for *R. solanacearum* which was a pathogen. As nano-silver had antibacterial effects, it could protect the seeds from pathogens, thus increasing germination rates. In sample T3, seeds were soaked in nano-silver and biofertilizer, which contained UPMR bio1 and UPMR bio2. Since the MICs for UPMR bio1 and UPMR bio2 were 40 mg l^−1^, soaking the seeds with 40 mg l^−1^ silver could inhibit the activity of beneficial microbes. For sample T4, where seeds were also soaked with AgNPs, the silver inhibited the activity of *R. solanacearum,* resulting in 100% germination. In samples T3 and T4, nano-silver at 40 mg l^−1^ inhibited the growth of Gram-negative beneficial microbes (UPMR bio1 and UPMR bio2) and pathogen *R. Solanacearum*, respectively.

In sample T5, containing *R. solanacearum* and biofertilizer, the lowest rate of seed germination (75%) was recorded. It was possible that there was an interaction between AgNPs, beneficial microbes, and the pathogen in this treatment, leading to reduced germination. For samples T3 and T4, 100% of the seeds germinated, with AgNPs only interacted with UPMR bio1 and UPMR bio2 in T3, and only with *R. solanacearum* in T4. As silver had to react with both the beneficial microbes and the pathogen to inhibit their activity/growth in sample T5, a lower amount of silver might have been available to react with the seeds.

## Discussion

Nano-silver, acting as a bactericide with particle size of 16 ±  6 nm, inhibited the activity of both Gram-positive and Gram-negative bacteria; however, the MICs of nano-silver were not the same among different strains of bacteria. Nano-silver applied to seeds at 40 mg l^−1^ could protect them from the pathogen *R. solanacearum*.

### Characterization of nano-silver bactericides

The purity of the NPs in solution was important, since low quality preparations might cause interactions with impurities, altering relative efficacies and affecting conclusions (Odum, 2007). Different morphologies have different surface areas to interact with microbes, resulting in different antibacterial efficiencies. Small-sized NPs have the potential to enter the cell membrane of bacteria (El-Kheshen & El-Rab, 2012). Silver NPs were spherical, giving them a high surface area to volume ratio and a stable morphology. Such factors were considered to enhance the efficacy and consistency of the experimental results, respectively. Krutyakov et al. (2008) and Xu et al. (2006) reported that spherical silver NPs were more stable and had a higher antibacterial activity compared to other shapes. The spherical shape of NPs resulted in a high surface area to volume ratio, which enabled them to pass through bacterial or plant cell membranes, killing the cell. Shameli et al. (2012) and Lkhagvajav et al. (2011) also reported that silver NPs with the sizes of 10–20 nm had the ability to inhibit bacterial activity. El-Kheshen & El-Rab (2012) stated that the antibacterial effect of nano-silver depended strongly on the size and the shape of the particles.

### MIC of nano-silver on selected bacteria

Several factors can influence the antibacterial activity of AgNPs, such as the chemical properties of NPs and the type of bacteria. The cell wall in bacteria is a layer outside the cell membrane which is made of peptides, known as peptidoglycans. Gram-positive bacteria have thicker cell walls compared to Gram-negative bacteria. In theory, NPs could enter bacteria through their cell walls (Theivasanthi & Alagar, 2011). Based on the findings of other researchers, there are two possible explanations to explain the differential interaction of NPs with bacterial cells, based on their Gram staining. Firstly, experimental studies on the cell surface of Gram-negative bacteria showed higher negative charges compared to Gram-positive bacteria. If NPs have a net positive charge, they will interact more readily with Gram-negative bacteria and exert a greater antibacterial activity than with Gram-positive bacteria (Chung et al., 2004). Secondly, Shahrokh & Emtiazi (2009) showed that AgNPs act as a catalyst in Gram-negative bacteria, influencing oxidation activity at higher concentrations, while in Gram-positive bacteria they are more effective at lower concentrations. These two possibilities might explain the higher MICs of nano-silver for Gram-negative bacteria compared with Gram-positive bacteria in our study. The exact mechanisms leading to the antibacterial effects of NPs are not fully understood; however, many theories have been put forward. A possible mechanism is that the NPs anchor themselves to the cell wall of bacteria and penetrate into the cell, making structural changes in the cell membrane that leads to cell death. Another possible mechanism is the formation of free radicals by some of the NPs, which create pores in the bacteria and leads to cell damage and eventually death. The other proposed mechanism is that silver can react with sulfur and phosphorus in the bacteria and form detrimental compounds which damage DNA and kills the cells (Prabhu & Poulose, 2012).

Odum (2007) demonstrated that in order to understand the antibacterial mechanism of silver NPs, the first step is to shed light on how bacteria and viruses live and grow. Bacteria use enzymes to metabolize oxygen. Silver ions cripple these enzymes, stop this metabolism, and kill cells. Viruses grow by attacking living cells and as they cannot function or reproduce outside the host cell, they are also reliant on oxygen metabolizing enzymes (Mishra & Kumar, 2009). Other studies have shown that monovalent silver ions (Ag^+^) can replace thiol (S-H) groups on the surface of cell membranes. This replacement disables the production of proteins required by the cell, leading to eventual death. Feng et al. (2000), reporting that nano-silver particles influence Gram-negative and Gram-positive bacteria, also pointed out that they not only attach to the surface of the cell but also enter the cell and collapse the cell wall.

### Effect of nano-silver bactericide on seed germination

The main plant physiological indices of the toxic effect of NPs are seed germination percentage, root elongation, biomass, and number of leaves (Yang, Cao & Rui, 2017). There is a possibility that NPs, such as Au, Ag, TiO_2_, CuO, and ZnO, can penetrate the seed coat causing an enhancement of water uptake by seeds, which results in early seed germination (Lei et al., 2008). Karimi et al. (2012) compared AgNPs with the fungicide carboxitiram and found that NPs with a size of 50 nm could protect seeds from pathogens. Our results with sample T4, containing 40 mg l^−1^ nano-silver and *R. solanacearum,* proved that silver had the ability to protect seeds from a pathogen, and even improve the rate of germination. Silver can inhibit the growth of bacteria and it is toxic for both Gram-negative and Gram-positive bacteria (Jung et al., 2010; Shahrokh & Emtiazi, 2009). Aquaporins are channel proteins present in the plasma and intracellular membranes of plant cells, where they facilitate the transport of water and small neutral solutes affecting seed germination. Silver NPs could also affect the aquaporin genes, increasing water uptake. Silver NPs improved the rate of seed germination in corn (*Zea mays*), watermelon (*Citrullus lanatus*), zucchini (*Cucurbita pepo*; Almutairi & Alharbi, 2015), ryegrass, barley, flax (El-Temsah & Joner, 2012), *Boswellia ovalifoliata* (Savithramma, Ankanna & Bhumi, 2012) and tomato (Khodakovskaya et al., 2009). Enhanced rates of germination with NPs were also observed for CuO in maize (Wang, Westerhoff & Hristovski, 2012), MWCNTs in zucchini (Stampoulis, Sinha & White, 2009), and mixed nano TiO_2_ and nano SiO_2_ in soybean (Lu et al., 2002). Silver NPs can also enhance the activity of antioxidant enzymes, superoxide dismutase (SOD) and catalase (CAT; Zheng et al., 2005). NPs can penetrate the seed coat and improve the rate of seed germination by enhancing water absorption, increasing nitrate reductase levels, promoting seed antioxidant systems, reducing antioxidant stress by decreasing H_2_O_2_, and finally increasing the activity of some enzymes such as SOD and glutathione peroxidase (Lei et al., 2008). Results from Savithramma, Ankanna & Bhumi (2012), Khodakovskaya et al. (2009), and Almutairi & Alharbi (2015) were in agreement with our results showing that silver NPs could increase seed germination percentage. In sample T5, silver NPs interacted with microbes (beneficial and pathogen) and there were likely not sufficient AgNPs available to attach to the seeds and play a positive role in improving the rate of seed germination. Silver NPs are capable of attaching themselves to the surface of cell membranes and disrupting permeability and respiration. It has been speculated that the binding of NPs to microorganisms depends on the available surface area for interaction. Silver NPs are capable of interacting with microorganisms, and when the volume of silver NPs reduces, larger surface area will be available to interact with microorganisms. Similar results were reported by Humberto et al. (2009) and Sondi & Salopek-Sondi (2004), stating that microorganisms were attached to the surface of silver NPs.

Even though the concentrations of NPs in the environment are lower than toxic levels, continuous use of NPs in agriculture can cause critical levels to accumulate. Since soil is considered as the final sink for NPs, they can enter the human food chain through plants. Therefore, it is essential to determine the amount of NPs which can protect plants from pathogens and diseases without harmful effects on the environment and human health (Yang, Cao & Rui, 2017). The risk of using nanotechnology in agriculture is poorly understood and requires research (Wickson, Nielsen & Quist, 2011).

## Conclusions

MIC results showed that nano-silver bactericides, as spheres with an average size of 16 ± 6 nm, inhibited the activity of bacteria (beneficial microbes and a pathogen) at levels of 12–40 mg l^−1^. Higher concentrations of nano-silver were needed to inhibit Gram-negative bacteria compared to Gram-positive bacteria. Bacteria classified as both NFB and PSB were also more resistant to Ag bactericide. Bacteria classified as Gram-negative and having both N_2_ fixing and phosphate solubilizing capabilities could be recommended for use as biofertilizer, since nano-silver inhibited their growth and activity at higher concentration (40 mg l^1^) than other beneficial microbes. Results also showed that nano-silver enhanced the rate of seed germination by protecting them from a pathogen. It was also observed that for increasing amounts of bacteria (beneficial microbes and pathogen), the concentration of nano-silver should be increased to get better seed germination percentage. Overall, AgNPs with sizes of 10–16 nm and a spherical shape could be used as a priming agent to protect seeds from pathogens and improve the rate of seed germination.

##  Supplemental Information

10.7717/peerj.6418/supp-1 File S1 Treatments and seed germination among all treatments Each data presents the percentage of seed germination among the different treatments.

10.7717/peerj.6418/supp-2 File S2 Statistical test for the effect of nano silver on seed germination A comparison of seed germination between different treatment.

## Additional Information and Declarations

### Data Availability

The following information was supplied regarding data availability: The raw data are available in a Supplemental File.

## References

[ref-1] Almutairi ZM, Alharbi A (2015). Effect of nano silver nano particles on seed germination of crop plant. International Journal of Nuclear and Quantum Engineering.

[ref-2] Chung YC, Su YP, Chen CC, Jia G, Wang HL, Wu JG, Lin JG (2004). Relationship between antibacterial activity of chitosan and surface characteristics of cell wall. Acta Pharmacologica Sinica.

[ref-3] Colman BP, Arnaout CL, Anciaux S, Gunsch CK, Hochella Jr MF, Lowry GV, McGill BM, Reinsch BC, Richardson CJ, Unrine JM (2013). Low concentrations of silver nanoparticles in bio solids cause adverse ecosystem responses under realistic field scenario. PLOS ONE.

[ref-4] Deberdt P, Guyot J, Coranson-Beaudu R, Launay J, Noreskal M, Riviére P, Vigné F, Laplace D, Lebreton L, Wicker E (2014). Diversity of *Ralstonia solanacearum* in french Guiana Expands knowledge of the emerging ecotype. Phytopathology.

[ref-5] El-Kheshen A, El-Rab SFG (2012). Effect of reducing and protecting agents on size of silver nanoparticles and their anti-bacterial activity. Der Pharma Chemica.

[ref-6] El-Temsah YS, Joner EJ (2012). Impact of Fe and Ag nano particles on seed germination and differences in bioavailability during exposure in aqueous suspension and soil. Journal of Environmental Toxicology.

[ref-7] Ericksen PJ, Ingram JS, Liverman DM (2009). Food security and global environmental change emerging challenges. Environmental Science & Policy.

[ref-8] Feng LQJ, Chen GQ, Cui FZ, Kim TN, Kim JO (2000). A mechanistic study of the antibacterial effect of silver ions on *Escherichia coli* and *Staphylococcus aureus*. Journal of Biomedical Materials Research.

[ref-9] Habib SH, Kausar H, Saud HM (2016). Plant growth-promoting rhizobacteria enhance salinity stress tolerance in okra through ROS-scavenging enzymes. BioMed Research International.

[ref-10] Hao Y, Cao X, Ma C, Zhang Z, Zhao N, Ali A, Hou T, Xing Z, Zhuang J, Wu S, Xing B, Zhang Z, Rui Y (2017). Potential application and antifungal activities of engineered nanomaterials against gray mold disease agent *Botrytis cinerea* on rose petals. Frontier in Plant Science.

[ref-11] Hojjat SS, Hojjat H (2015). Effect of nano silver on seed germination and seedling growth in Fenugreek seed. International Journal of Food Engineering.

[ref-12] Holden PA, Schimel JP, Godwin HA (2014). Five reasons to use bacteria when assessing manufactured nanomaterial environmental hazards and fates. Current Opinion in Biotechnology.

[ref-13] Humberto L, Nilda V, Ayala N, Lilian DCIT, Padill CRG (2009). Bactericidal effect of silver nanoparticles against multidrug resistant bacteria. World Journal of Microbiology Biotechnology.

[ref-14] Jung J-H, Kim S-W, Min J-S, Kim Y-J, Lamsal K, Kim KS, Lee YS (2010). The effect of nano-silver liquid against the white rot of the green onion caused by *Sclerotium cepivorum*. Mycobiology.

[ref-15] Karimi N, Minaei S, Almassi M, Shahverdi AR (2012). Application of silver nano-particles for protection of seeds in different soils. African Journal of Agriculture Research.

[ref-16] Khodakovskaya M, Derivishi E, Mohammad M, Xu Y, Li Z, Watanabe F, Bris AS (2009). Carbon nanotubes are able to penetrate plant seed coat and dramatically affect seed germination and plant growth. ACS Nano.

[ref-17] Krutyakov YA, Kudrinskiy AA, Olenin AY, Lisichkin GV (2008). Synthesis and properties of silver nanoparticles: advances and prospects. Russian Chemical Reviews.

[ref-18] Lee PC, Meisel D (1982). Adsorption and surface-enhanced Raman of dyes on silver and gold sols. Journal of Physical Chemistry.

[ref-19] Lei Z, Mingyu S, Xiao W, Chao L, Chunxiang Q, Liang C, Fashui H (2008). Antioxidant stress is promoted by nano-anatase in spinach chloroplasts under UV-B radiation. Biological Trace Element Research.

[ref-20] Lkhagvajav N, Yasa I, Celik E, Koizhaiganova M, Sari O (2011). Antimicrobial activity of colloidal silver nanoparticles prepared by Sol-Gel method. Journal of Nnanomaterials and Biostructures.

[ref-21] Lu C, Zhang C, Wen J, Wu G, Tao M (2002). Research of the effect of nano-meter materials on germination and growth enhancement of Glycine max and its mechanisms. Soybean Science.

[ref-22] Mishra VK, Kumar A (2009). Impact of metal nanoparticles on the plant growth promoting rhizobacteria. Journal of Nanomaterials & Biostructures.

[ref-23] Odum LM (2007). Effect of silver nanoparticles on tomato plant and development of a plant monitoring system. Master Thesis.

[ref-24] Prabhu S, Poulose EK (2012). Silver nanoparticles: mechanism of antimicrobial action, synthesis, medical applications, and toxicity effects. International Nano Letters.

[ref-25] Rui M, Ma C, Hao Y, Guo J, Rui Y, Tang X, Zhao Q, Fan X, Zhang Z, Hou T, Zhu S (2016). Iron oxide nanoparticles as a potential iron fertilizer for peanut (*Arachis hypogaea*). Frontiers in Plant Science.

[ref-26] Rui M, Ma C, White JC, Yi Hao, Wang Y, Tang X, Yang J, Jag F, Ali A, Rui Y, Cao W, Chen G, Xing B (2018). Metal oxide nanoparticles alter Peanut (Arachis hypogaea L.) Physiological response and reduce nutritional quality: a life cycle study. Environmental Science: Nano.

[ref-27] Savithramma N, Ankanna S, Bhumi G (2012). Effect of nanoparticles on seed germination and seedling growth of *Boswellia ovalifoliolata* an endemic and endangered medicinal tree taxon. Nano Vision.

[ref-28] Shahrokh S, Emtiazi G (2009). Toxicity and unusual biological behavior of nano silver on gram positive and negative bacteria assayed by microtiter-plate. European Journal of Biological Sciences.

[ref-29] Shameli K, Bin Ahmad M, Jaffar Al-Mulla EA, Ibrahim NA, Shabanzadeh P, Rustaiyan A, Abdollahi Y, Bagheri S, Abdolmohammadi SMS, Usman ZM (2012). Green biosynthesis of silver nanoparticles using *Callicarpa maingayi* stem bark extraction. Molecules.

[ref-30] Simonin M, Richaume A (2015). Impact of engineered nanoparticles on the activity, abundance, and diversity of soil microbial communities: a review. Environmental Science & Pollution Research.

[ref-31] Sondi I, Salopek-Sondi B (2004). Silver nanoparticles as antimicrobial agent: a case study on E. coli as a model for Gram-negative bacteria. Journal of Colloid and Interface Science.

[ref-32] Sotiriou GA, Pratsinis SE (2011). Engineering nano silver as an antibacterial, biosensor and bioimaging material. Current Opinion in Chemical Engineering.

[ref-33] Stampoulis D, Sinha SK, White JC (2009). Assay—dependent phytotoxicity of nanoparticles to plants. Environmental Science & Technology.

[ref-34] Theivasanthi T, Alagar M (2011). Anti-bacterial studies of silver nanoparticles.

[ref-35] Wang Y, Westerhoff P, Hristovski KD (2012). Fate and biological effects of silver, titanium dioxide, and C_60_ (fullerene) nanomaterials during simulated waste water treatment processes. Journal of Hazardous Materials.

[ref-36] Wickson F, Nielsen NK, Quist D (2011). Nano and the environment: potential risks, real uncertainties and urgent issues. Biosafety Brief, 2011.

[ref-37] Xu R, Wang D, Zhang J, Li Y (2006). Shape-dependent catalytic activity of silver nano particles for the oxidation of styrene. Chemistry.

[ref-38] Yang J, Cao W, Rui Y (2017). Interactions between nanoparticles and plants: phytotoxicity and defense mechanisms. Journal of Plant Interactions.

[ref-39] Zarei M, Jamnejad A, Khajehali E (2014). Antibacterial effect of silver nanoparticles against four foodborne pathogens. Jundishapur Journal of Microbiology.

[ref-40] Zheng L, Hong F, Lu S, Liu C (2005). Effect of nano-TiO2 on strength of naturally aged seeds and growth of spinach. Biological Trace Element Research.

