# Supplementary material for: Minimum inhibitory concentration of nano-silver bactericides for beneficial microbes and its effect on Ralstonia solanacearum and seed germination of Japanese Cucumber (Cucumis sativus)
Source: PeerJ. 2019 Mar 20;7:e6418. doi: 10.7717/peerj.6418 (PMC6431134; doi:10.7717/peerj.6418)
Supplement: File S1 — Each data presents the percentage of seed germination among the different treatments. [file peerj-07-6418-s001.pdf]

Raw Data used for seed germination

Treatments	Replication	Seed germination
T1	R1	75
T1	R2	100
T1	R3	75
T1	R4	100
T2	R1	100
T2	R2	100
T2	R3	100
T2	R4	100
T3	R1	100
T3	R2	100
T3	R3	100
T3	R4	100
T4	R1	100
T4	R2	100
T4	R3	100
T4	R4	100
T5	R1	75
T5	R2	100
T5	R3	75
T5	R4	50
