# Supplementary material for: Minimum inhibitory concentration of nano-silver bactericides for beneficial microbes and its effect on Ralstonia solanacearum and seed germination of Japanese Cucumber (Cucumis sativus)
Source: PeerJ. 2019 Mar 20;7:e6418. doi: 10.7717/peerj.6418 (PMC6431134; doi:10.7717/peerj.6418)
Supplement: File S2 — A comparison of seed germination between different treatment. [file peerj-07-6418-s002.docx]

Appendix

The ANOVA Procedure

| Class Level Information | | |
| --- | --- | --- |
| Class | Levels | Values |
| Trt | 5 | T1 T2 T3 T4 T5 |

| Number of Observations Read | | 20 |  |  |  |
| --- | --- | --- | --- | --- | --- |
| The ANOVA Procedure    Dependent Variable: germination | |  |  |  |  |
| Source | DF | Sum of Squares | Mean Square | F Value | Pr > F |
| Model | 4 | 2000.000000 | 500.000000 | 4.00 | 0.0210 |
| Error | 15 | 1875.000000 | 125.000000 |  |  |
| Corrected Total | 19 | 3875.000000 |  |  |  |

| R-Square | Coeff Var | Root MSE | germination Mean |
| --- | --- | --- | --- |
| 0.516129 | 12.08685 | 11.18034 | 92.50000 |

| Source | DF | Anova SS | Mean Square | F Value | Pr > F |
| --- | --- | --- | --- | --- | --- |
| Trt | 4 | 2000.000000 | 500.000000 | 4.00 | 0.0210 |

The ANOVA Procedure

Dependent Variable: Y

| Source | DF | Sum of Squares | Mean Square | F Value | Pr > F |
| --- | --- | --- | --- | --- | --- |
| Model | 4 | 1.30024561 | 0.32506140 | 4.39 | 0.0151 |
| Error | 15 | 1.11031108 | 0.07402074 |  |  |
| Corrected Total | 19 | 2.41055670 |  |  |  |

| R-Square | Coeff Var | Root MSE | Y Mean |
| --- | --- | --- | --- |
| 0.539396 | 19.80272 | 0.272068 | 1.373890 |

| Source | DF | Anova SS | Mean Square | F Value | Pr > F |
| --- | --- | --- | --- | --- | --- |
| Trt | 4 | 1.30024561 | 0.32506140 | 4.39 | 0.0151 |
